# Ultra-Processed Food Addiction: A Research Update

**DOI:** 10.1007/s13679-024-00569-w

**Published:** 2024-05-18

**Authors:** Erica M. LaFata, Kelly C. Allison, Janet Audrain-McGovern, Evan M. Forman

**Affiliations:** 1 Center for Weight, Eating, and Lifestyle Science, Drexel University, 3201 Chestnut Street, Philadelphia, PA 19104 United States; 2 Center for Weight and Eating Disorders, Department of Psychiatry, Perelman School of Medicine, University of Pennsylvania, 3535 Market Street, Philadelphia, PA 19104 United States; 3 Department of Psychiatry, Perelman School of Medicine, University of Pennsylvania, 3535 Market Street, Philadelphia, PA 19104 United States; 4 Department of Psychological and Brain Sciences, Drexel University, 3201 Chestnut Street, Philadelphia, PA 19104 United States

**Keywords:** Ultra-processed foods, Ultra-processed food addiction, Compulsive overeating, Obesity treatment outcomes

## Abstract

**Purpose of Review:**

Detail recent advancements in the science on ultra-processed food (UPF) addiction, focusing on estimated prevalence rates and emerging health disparities; progress towards identifying biological underpinnings and behavioral mechanisms; and implications for weight management.

**Recent Findings:**

Notable developments in the field have included: (1) estimating the global prevalence of UPF addiction at 14% of adults and 15% of youths; (2) revealing health disparities for persons of color and those with food insecurity; (3) observing altered functioning across the brain-gut-microbiome axis; (4) providing early evidence for UPF withdrawal; and (5) elucidating poorer weight management outcomes among persons with UPF addiction.

**Summary:**

The breadth of recent work on UPF addiction illustrates continued scientific and public interest in the construct and its implications for understanding and treating overeating behaviors and obesity. One pressing gap is the lack of targeted interventions for UPF addiction, which may result in more optimal clinical outcomes for this underserved population.

## Introduction

Over the past 40 years, global rates of overweight and obesity have increased substantially [1], leading researchers to explore contributing mechanisms. While numerous biological (e.g., genetic, hormonal) and behavioral (e.g., sedentary lifestyle) factors are known to be associated with obesity [2], one of the key systemic changes in our environment that has paralleled the timeline of rising obesity rates has been the exponential increase of ultra-processed foods (UPFs) in our food system since the 1980s [3, 4]. UPFs are not found in nature and have been intentionally created to be hyper-palatable through the addition of added fats, refined carbohydrates (e.g., white flour, sugar), and/or salt [5, 6]. Examples of UPFs include pastries, candy, packaged snacks, fast foods, and sugar-sweetened beverages [6]. Rigorous, epidemiological research has soundly implicated UPFs in not only obesity but also in an array of physical health conditions. As detailed in a recent review [7], four prospective cohort studies (*n*s = 11,898-44,551; mean follow-up = 6.6–19 years) have consistently observed that persons reporting the highest versus lowest quartile of UPF intake at baseline had a 25–62% increased hazard for all-cause mortality, and, relatedly, numerous prospective and cross-sectional studies have linked increased UPF intake with elevated rates and/or complications of diet-related conditions (e.g., cardiovascular disease, type 2 diabetes).

While it has long been possible to create homemade versions of popular UPFs (e.g., homemade chocolate chip cookies), some researchers have noted that the uptick in rates of obesity and diet-related diseases have occurred on a similar timeline as industry-driven changes in the global food system [8]. One such change was the increased development and distribution of UPFs that followed after Kraft Foods and General Foods were acquired by Phillip Morris – one of the largest tobacco companies worldwide [9•, 10]. Released industry documents have revealed that the same tobacco industry leaders admitted to applying the techniques used to enhance the addictive properties of tobacco products to the development of UPFs (e.g., use of additives to enhance flavor, texture, and visual appeal) in order to maximize profits [9•]. As such, the direct role of UPFs in driving the obesity epidemic may be at least partially explained by the tobacco industry’s involvement in designing UPFs to be highly reinforcing, using practices like those used for maximizing the addictive potential of tobacco products.

Notably, preclinical and human studies have demonstrated that repeated intake of UPFs triggers addiction-like biological (e.g., dopaminergic sensitization) and behavioral (e.g., withdrawal, use despite consequences) responses, whereas consumption of naturally occurring foods like fruits, vegetables, and meats have demonstrated limited or no associations with indicators of addiction (for a review of the evidence for the addictive properties of UPFs, see [11••]). This differentiation has been attributed to UPFs sharing pharmacokinetic properties with addictive substances, namely containing artificially high doses of rewarding ingredients (e.g., added sugars) that are rapidly absorbed by the system [12]. Strikingly, researchers recently argued that UPFs meet the scientific criteria used to define tobacco products as addictive [13].

Evidence for the addictive potentials of UPFs has been coupled with empirical investigations of the plausibility of UPF addiction as a novel clinical presentation, which has been most commonly operationalized by adapting the diagnostic criteria for substance-use disorders (SUDs) to the intake of UPFs (for a review, see [14••]). While individuals can experience problematic eating behaviors with a wide array of foods, the presentation of UPF addiction is specific to exhibiting diagnostic features of SUDs specifically with UPFs (akin to alcohol-use disorder being specific to experiencing diagnostic indicators of SUDs with alcoholic beverages) [14••]. The focus on UPFs reflects the strong science that UPFs trigger addictive responses in a manner that other foods do not and importantly addresses a main critique of the construct (distinguishing UPF addiction from the homeostatic need to eat) [11••]. Evaluating the evidence for UPF addiction also requires acknowledgement of the key limitations of the literature to date, such as the lack of research exploring the addictive ingredient/attribute of UPFs (refined carbohydrates vs. added fats vs. both) [15, 16] and the limited studies clarifying the shared and distinct features of UPF addiction versus existing eating disorders [17, 18]. In addition, UPF addiction is not a recognized clinical diagnosis in the DSM-5, and further investigation on this topic is needed to understand if this presentation may warrant consideration as a novel diagnostic category in the future.

As such, while this this review details recent advancements in the empirical understanding of UPF addiction, future research directions are also highlighted to underscore gaps in the existing literature. Specifically, the authors will describe: (1) global population prevalence estimates of UPF addiction and health disparities; (2) recent research on the biological underpinnings and behavioral features of UPF addiction; (3) implications of UPF addiction for weight management; and (4) key next steps for clinical research and treatment to understand UPF addiction both within the context of obesity and as a distinct clinical presentation.

## Current Prevalence Estimates of UPF Addiction and Evidence for Health Disparities

The Yale Food Addiction Scale (YFAS) measures remain the most common empirical approach for operationalizing UPF addiction [19]. The current version of the YFAS (YFAS 2.0) parallels the Diagnostic and Statistical Manual of Mental Disorders (5th edition; DSM-5) approach for diagnosing SUDs [20]. Specifically, individuals can meet for UPF addiction by endorsing at least two of the 11 symptoms of SUDs with respect to their intake of UPFs (e.g., use despite negative consequences, persistent unsuccessful attempts to cut down, withdrawal), plus clinically significant impairment (e.g., interference with role obligations) or psychological distress [20]. Two recent systematic reviews of 281 studies across 36 countries estimated the prevalence of UPF addiction in the general population as 14% in adults [21]• and 15% in youths [22•]. The rate of UPF addiction among adults is highly similar to the prevalence of SUDs where the addictive substance is legal, such as alcohol-use disorder (14%) and tobacco-use disorder (18%) [23, 24]. However, the prevalence of UPF addiction in youths is striking and unprecedented [25••]. Across the lifespan, individuals with obesity seem to be at higher risk for UPF addiction, with prevalence estimated at 28% of adults and 19% of youths with obesity [21•, 22•]. While UPF addiction may be more common among persons with obesity, the observed degree of overlap demonstrates that the two conditions are not synonymous and should not be conflated.

Progress towards understanding the demographic correlates of UPF addiction has revealed significant health disparities [25••]. A nationally representative sample of the United States revealed that the prevalence of UPF addiction was substantially higher among individuals who identified with an underrepresented racial or ethnic group (Black: 16.8%; Hispanic: 32.3%) compared to those who identified as non-Hispanic and White (11.7%) [26]. Further, UPF addiction was more strongly associated with obesity for individuals who reported lower versus higher household incomes [26]. In addition, food insecurity has been associated with a 21–56% greater risk of meeting criteria for UPF addiction [27, 28] and persons with lower household incomes are more likely to have obesity if they meet criteria for UPF addiction [26]. These health disparities may be largely driven by two key factors. First, food industry documents have revealed that UPFs are disproportionately marketed to persons of color using exploitative techniques taken from the playbook of Big Tobacco [29••]. Second, the accessibility and affordability of rewarding, calorie-dense UPFs in our environment makes them, understandably, a highly enticing choice for individuals with limited time and/or financial resources or those experiencing prolonged stress.

## Recent Research on the Biological Underpinnings of UPF Addiction: A Focus on Alterations in the Brain-Gut-Microbiome Axis

The foundation of biological evidence for the addictive potentials of UPFs comes from prior neuroimaging studies that have observed similar reward-related neural responses associated with UPFs and addictive substances [30, 31]. Further, individuals with elevated symptoms or who meet criteria for UPF addiction exhibit neural responses to UPFs similar to those observed among those with a SUD for their addictive substance of choice (e.g., greater anticipatory reward, reduced consummatory reward, enhanced functional connectivity across reward processing regions) [32–34]. A 2021 review provides a detailed account of the neurobiological research supporting the plausibility of UPF addiction [35]. Further, a 2023 review summarizes 16 studies that have observed associations between UPF addiction and blood biomarkers (e.g., glucose, insulin, leptin, ghrelin) and critically raises methodological limitations that require attention in future work (e.g., small sample sizes, variation in comorbidities) [36]. Thus, the current section will focus on another emerging area of studies exploring the biological underpinnings of UPF addiction—alterations in the brain-gut-microbiome axis implicated in chronic UPF intake and UPF addiction.

Preclinical models can examine the causal consequences of UPF intake on the brain-gut-microbiome axis by introducing UPFs to rats without previous exposure. In rats prone to obesity, repeated intake of UPFs triggers a rapid upregulation in calcium-permeable AMPA receptors in the nucleus accumbens, which is characteristic of addictive substances and associated with increased cue-induced craving and drug-seeking behavior [37–40]. This UPF-induced alteration to the reward system notably preceded the onset of obesity [37, 39, 40]. Prolonged consumption of UPFs among obesity-prone rats has also been found to cause reduced excitability of nucleus accumbens core neurons, which is suggestive of altered dopaminergic reward responses and has been similarly demonstrated with chronic cocaine exposure [41, 42]. Another study demonstrated that prolonged UPF consumption alters the gut microbiome in a manner that creates an abundance of microbes implicated in obesity and metabolic disease [43]. Across these studies, addiction-like alterations were not found to occur when obesity-prone rats consumed their nutritionally balanced chow [38–43].

Collectively, this animal research provides convincing support for the direct and unique role that UPFs have in promoting obesity via their ability to alter the brain-gut-microbiome axis in a manner that increases craving and motivates continued UPF intake. However, unlike studies of rats, in which UPFs can be introduced for the first time and the causal effects on the brain-gut-microbiome axis can be observed, the potential to observe such effects in humans is limited by the high rate of typical UPF intake in the population. As such, preclinical models lay the groundwork for the translational research that can be feasible explored in humans.

Relatedly, human research has identified numerous alterations across the brain-gut-microbiome axis among individuals with UPF addiction. Chronically high levels of UPF intake among persons with UPF addiction has been associated with disrupted dopaminergic signaling (increased hedonic drive for UPFs), dysregulated hunger/satiety hormones (increased hunger, reduced satiety), and alterations to the gut microbiome related to obesity risk [44]. Similarly, women with versus without UPF addiction were more likely to exhibit gut dysbiosis, marked by an overabundance of microbes implicated in insulin resistance and greater propensity for obesity, and the presence of gut dysbiosis was also associated with increased neural connectivity in reward motivation regions [45]. Further, one study observed that individuals with UPF addiction and overweight/obesity were more likely to exhibit gut metabolomic patterns associated with high-risk traits for addictive disorders (e.g., emotion dysregulation, increased reward seeking), and the presence of this gut microbial signature mediated the positive relationship between UPF addiction and body mass index (BMI) [46].

Overall, there is emerging evidence for altered functioning across the brain-gut-microbiome axis among individuals with UPF addiction, which appear to be closely implicated in perpetuating compulsive eating behavior and/or increasing one’s risk for obesity.

## Recent Research on the Behavioral Features of UPF Addiction

UPF addiction has been most consistently operationalized by adapting the 11 DSM-5 behavioral criteria for diagnosing SUDs to the addictive-like intake of UPFs [14••]. One theme of recent studies and scientific reviews has been to consider if and how the behavioral features of UPF addiction represent a distinct clinical presentation from existing eating disorders [47–49]. Importantly, overlap exists between several of the 11 DSM-5 diagnostic indicators of SUDs and problematic overeating behaviors, such as consuming more than intended, wanting to cut down but being unable to do so, and experiencing cravings. In binge-type eating disorders, overlapping behavioral features with addictive disorders can be more substantial, extending to symptoms like continued consumption despite negative physical/psychological consequences, interference with role obligations, and significant time spent obtaining, consuming, and recovering from the effects of the food/substance. As may be expected, the prevalence of UPF addiction among individuals with binge-type eating disorders is high (48–55% in a global meta-analysis [21•]).

Yet, most individuals with UPF addiction do not have an eating disorder, as defined by the DSM-5, as the population prevalence rate of UPF addiction is 14% [21•] and of a binge-type eating disorder is 1.0-2.8% [50]. The increased prevalence of UPF addiction compared to binge-type eating disorders may reflect the wider range of problematic behaviors encompassed by an addiction framework. While binge consumption is one presentation of addiction (e.g., binge drinking, chain smoking), many individuals meet criteria for a SUD by using their substance of choice in a grazing pattern (e.g., smoking cigarettes throughout the day). In parallel, while binge-type eating disorders are characterized by 2-hour episodes where an objectively large amount of food is consumed, UPF addiction may present as binge episodes and/or grazing (e.g., eating UPFs frequently throughout the day in response to persistent cravings). Importantly, research is needed to characterize common presentations of UPF addiction, as no data exist to speak to the frequency of addictive-like UPF intake occurring in binge episodes versus grazing patterns.

Two key differences in the presentation of UPF addiction, relative to binge-type eating disorders, are (1) the specificity of the addictive-like eating behaviors occurring with UPFs; and (2) the absence of a defined threshold for the quantity of food that must be consumed. Importantly, while no SUDs have a set threshold for how much of an addictive substance must be taken to meet diagnostic criteria, clinical judgment is used to ensure that individuals are using addictive substances to the extent that their use leads to impairment and/or distress. Similarly, while no set quantity of UPF intake is required to meet criteria for UPF addiction, the clinical presentation is intended to operationalize problematic overeating patterns with UPFs.

Research is needed to examine if the quantity of UPF intake has clinical relevance among individuals with UPF addiction. Similar consideration has been given to whether binge episodes (in the context of binge-type eating disorders) need to involve an objectively large amount of food, in light of prior studies finding stronger associations between increased symptoms of disordered eating and one’s experience of loss of control overeating versus the size of the binge [51, 52]. In addition, following bariatric surgery, loss of control eating can re-emerge, but less commonly are patients able to consume objectively large amounts of food. Akin to ongoing discussions of whether this post-operative, loss of control eating should be classified as a binge-type eating disorder [53], research is warranted to inform the assessment of addictive-like UPF intake among individuals with conditions that physically limit their food intake. Further, although individuals with anorexia nervosa endorse high levels of UPF addiction on the self-report YFAS measures [54, 55], these estimates have been recently discussed as likely false positives [14••, 56•]. Presently, a clinician-guided tool for diagnosing UPF addiction is in development, which will likely clarify UPF addiction rates among persons with restrictive-type eating disorders.

In a similar vein of evaluating UPF addiction as a distinct clinical presentation, a second emerging trend in behavioral research is the evaluation of whether withdrawal can occur with UPFs [57, 58]. Withdrawal is a core diagnostic indicator of SUDs that is not acknowledged by theoretical perspectives of obesity or eating disorders. As such, researchers have suggested that scientific evidence for the plausibility of UPF withdrawal would also support the clinical utility of UPF addiction as a unique presentation of problematic overeating [58]. While tolerance is also a unique feature of SUDs, less research has systematically assessed tolerance to UPFs, given the high levels of UPF consumption in the population, and thus withdrawal has been the mechanism of focus (for a review of neurobiological evidence for tolerance to UPFs, see [57]).

Early evidence for the plausibility for UPF withdrawal came from preclinical models, which observed that rats exhibited withdrawal-like symptoms (e.g., anxiety, teeth chattering) when UPFs or sugar (an ingredient often added to UPFs) were removed from their diets [59]. These findings sparked investigations of UPF withdrawal in humans, and though all human studies to date have relied on retrospective self-report, the preliminary data is similarly suggestive of its plausibility [58]. For instance, qualitative studies have revealed that both adults and youths describe experiencing physical (e.g., headaches, fatigue) and psychological (e.g., irritability, preoccupation with cravings) symptoms when trying to reduce UPFs that parallel features of nicotine withdrawal [60–62]. Further, the withdrawal symptom on the YFAS measures has been endorsed by 18.5–29.7% of adults and 18.9% of youths in general population samples [20, 26, 63].

Systematic investigations of UPF withdrawal in humans has been facilitated by the recent development of the Highly Processed Food Withdrawal Scale (ProWS) [64] and its adaptation designed for parents to report on their children’s UPF withdrawal symptoms (ProWS-C) [65]. (In this scale, “highly processed food” is synonymous with UPF.) The validation studies for both the ProWS and ProWS-C measures demonstrated that adults and youths, respectively, reported physical (e.g., headaches) and psychological (e.g., irritability, preoccupation) symptoms reminiscent of tobacco withdrawal during their most recent attempt to cut down on UPFs [64, 65]. Both samples indicated that these withdrawal-like symptoms were most intense 2–5 days after reducing UPFs and decreased in intensity over the following 7–14 days, which parallels the time course of withdrawal syndromes for addictive substances [64, 65]. Further, in both adults and youths, increased UPF withdrawal symptoms were associated with self-reported history of weight cycling and successful dietary adherence above and beyond BMI. Critically, methodologically rigorous, prospective techniques (e.g., randomized controlled trials, ecological momentary assessment) are needed to validate the presence, features, and time course of UPF withdrawal. Nonetheless, evidence to date from preclinical and human studies supports the plausibility of UPF withdrawal and suggests its clinical relevance to weight management and adherence to dietary recommendations.

## Emerging Evidence for the Clinical Utility of UPF Addiction within Overweight or Obesity

In the past 5 years, evidence has grown for the clinical relevance of UPF addiction among individuals with overweight or obesity. The most compelling finding to date resulted from a large, randomized controlled trial of 609 adults who participated in a 12-month behavioral weight loss intervention [66••]. Strikingly, of the 41 demographic and psychosocial factors examined, symptoms of UPF addiction at baseline was the single strongest psychosocial predictor of both treatment dropout and lower weight loss [66••]. Other baseline characteristics that were found to be less predictive of these outcomes than symptoms of UPF addiction (or not significant at all) included self-efficacy for dietary change, emotional eating, body dissatisfaction, social support for eating habits, perceived stress, quality of life, dietary restraint, depression, sleep quality, age, education, and race [66••]. Three other related studies yielded mixed findings on the association of UPF addiction with outcomes in obesity treatments [67–69], though null findings may have been partially attributable to limitations in sample size, treatment duration, and/or intervention approach [66••]. While replication is warranted, the recent findings from Fielding-Singh and colleagues [66••] convincingly illustrate that UPF addiction warrants assessment and intervention among persons seeking behavioral weight loss treatment.

The relevance of UPF addiction to weight management was further elucidated by Schulte and colleagues [70], who observed that adults with versus without UPF addiction reported gaining six times more weight during the first year of the COVID-19 pandemic (12.42 vs. 2.14 pounds, respectively). Further, those with UPF addiction reported consuming greater quantities of UPFs both before and during COVID-19 and endorsed greater distress related to their eating habits [70]. Though this study was cross-sectional, the findings are suggestive of the potential for UPF addiction to be a risk factor for weight gain, UPF overconsumption, and psychologically distressing eating behaviors.

Speaking to another facet of the relationship between UPF addiction and weight management, Leary and colleagues [71] reviewed eight studies that assessed the efficacy of pharmacological (naltrexone/bupropion, pexacerfont), bariatric surgery, and lifestyle (individual or group-based behavioral counseling, calorie restriction, and/or physical activity promotion) interventions for obesity for improving UPF addiction. Five of the eight studies observed improvements in UPF addiction among adults with obesity, in which the interventions included medications (naltrexone/bupropion and pexacerfont), bariatric surgery, and a behavioral weight loss intervention involving meal replacements [71]. Interestingly, the medications that led to improvements in UPF addiction have also demonstrated efficacy for decreasing cravings for addictive substances and treating SUDs (though naltrexone and bupropion are prescribed separately for those with an addiction) [72, 73].

From the small number of studies included in Leary and colleagues’ review [71], it is apparent that more research is needed to explore how existing interventions for overweight and obesity may have secondary benefits for improving UPF addiction. However, it is also evident from Fielding-Singh and colleagues’ [66••] data that exhibiting UPF addiction may substantially interfere with one’s ability to engage in weight management treatments or achieve weight loss. The complex, bidirectional relationship between UPF addiction and obesity treatments warrants attention in future research. One pressing and unexplored focus may be the identification of risk and resilience factors among those with UPF addiction that differentially predict who does versus does not achieve clinically meaningful weight loss through existing treatments.

## Next Steps for the Treatment of UPF Addiction

Table 1 summarizes the future directions for advancing the science on UPF stated earlier in this review, as well as the ideas for treating UPF addiction described in this section. At present, no evidence-based interventions have been specifically developed to treat UPF addiction. Yet, there are online self-help and community-based resources for individuals who self-identify as having a “food addiction,” which suggests there is an unaddressed need for scientifically informed clinical assessments and interventions for persons who exhibit addictive-like food intake. McKenna and colleagues [74] found that nearly all online support options aligned with the 12-step abstinence framework of community-based SUD treatments (e.g., Alcoholics Anonymous), which has been adapted as Overeaters Anonymous (OA). While OA does not use the term UPFs, the food plans and abstinence guidelines of OA emphasize abstinence from foods with sugar and refined carbohydrates, which would be classified as UPFs.

**Table 1:** Next steps to advance the science on ultra-processed food addiction

Clinical Assessment
1. Develop a clinician-guided interview for assessing and diagnosing UPF addiction (in progress)
2. Estimate the prevalence rates of UPF addiction in the general population and in clinical samples using the interview and compare estimates from prior studies using the YFAS measures
3. Utilize the clinician-guided interview to explore cultural differences in the presentation of UPF addiction
4. Characterize common presentations of UPF addiction to elucidate the frequency of addictive-like UPF intake occurring in binge episodes versus grazing patterns

Biobehavioral Research
1. Determine contributing factors to observed health disparities in UPF addiction by race, income, and food insecurity (e.g., targeted marketing campaigns)
2. Conduct longitudinal investigations in humans to understand the temporal relationships between changes in UPF intake and alterations in the brain-gut-microbiome axis
3. Use prospective, repeated-measures techniques, such as ecological momentary assessment, and randomized controlled trials to evaluate the presence and time course of UPF withdrawal
4. Evaluate the influence of addiction mechanisms on weight management treatments, such as whether early experiences of UPF withdrawal interfere with dietary adherence and motivate treatment dropout

Intervention Development
1. Conduct randomized controlled trials to evaluate and compare the feasibility and efficacy of abstinence-based and harm reduction treatments for UPF addiction
2. Explore individual characteristics that may inform when an abstinence-based versus harm reduction approach for treating UPF addiction may be more optimal
3. Identify risk and resilience factors that differentiate individuals with UPF addiction who do versus do not achieve desired outcomes in existing treatments for obesity
4. Test whether addressing addiction mechanisms in weight management interventions (e.g., coping with acute and prolonged withdrawal symptoms, pharmacology to regulate reward responsiveness) improves outcomes for individuals with obesity and UPF addiction

*UPF* Ultra-Processed Food, *YFAS* Yale Food Addiction Scale

Given the anonymous nature of OA, no randomized controlled trials have explored the program’s efficacy. The limited data on OA stems from case series and qualitative research, though these studies suggest benefits for binge-type eating disorders and obesity (for a review, see [75]). Potential benefits of OA’s abstinence-based approach to treating UPF addiction may be gleaned from a recent pilot trial of 103 adults, which demonstrated that a 10-to-14-week, group-based intervention emphasizing abstinence from UPFs led to significant reductions in symptoms of UPF addiction [76]. In addition, a case series and pilot trial have shown that the ketogenic diet, which severely limits sugars and refined carbohydrates, may also lead to reductions in symptoms of UPF addiction [77, 78]. Rigorous, randomized controlled trials are needed to evaluate if abstaining from UPFs is an appropriate dietary recommendation for the treatment of UPF addiction.

Some have questioned the long-term feasibility of abstinent approaches, and not all evidence-based treatments for SUDs prescribe abstinence. For example, harm reduction is an efficacious treatment for alcohol-use disorder that nuances being abstinent from high-risk use patterns (e.g., hard liquor, drinking when alone or in a negative mood state) and engaging in low-risk use patterns (e.g., having one glass of wine at a restaurant with friends) [79]. This approach has been particularly effective at preventing relapse among individuals who perceive alcohol to be integrated into their social circles in normative ways and can identify that their problematic drinking behaviors occur primarily in specific contexts [80]. The social acceptability of and abundant access to UPFs in our society may necessitate prioritization on evidence-based interventions that do not require abstinence from all UPFs (e.g., splitting a dessert with friends at a restaurant but not keeping pastries or packaged sweets in the home). Research is needed to determine whether a harm reduction approach is effective for treating UPF addiction, and if so, how it compares to recommendations to abstain from UPFs in terms of adherence and outcomes.

The notable finding that UPF addiction was the strongest psychosocial predictor of poorer engagement and outcomes in a behavioral weight loss program [66••] raises concern about how existing behavioral interventions may overlook, or even worsen, symptoms of UPF addiction among individuals with overweight or obesity. Commonly, behavioral weight loss treatments are based on the Diabetes Prevention Program, emphasizing dietary modification, increased physical activity, self-monitoring, and psychological influences on lifestyle changes (e.g., stimulus control, negative thought patterns) [81]. While the program recommends limiting high-calorie foods (often UPFs), dietary changes are centered on meeting a calorie goal that promotes weight loss, which allows for all foods to be consumed in moderation. The program acknowledges that individuals may have momentary lapses and longer-lasting relapses in meeting their calorie target [81], though no connections are made to how these setbacks may, for some people, stem from the intake of UPFs.

From an addiction standpoint, the intermittent intake of an addictive substance may increase the reinforcement that a person experiences, particularly for individuals with a SUD [82]. Similarly, for individuals with UPF addiction, the common behavioral weight loss principle of eating all foods in moderation, especially within a calorie target, may inadvertently promote UPF cravings and subsequent UPF intake (a lapse/relapse). In addition, features of addiction, such as withdrawal, may interfere with early dietary adherence and motivate treatment dropout for persons with UPF addiction. Future research is needed to understand whether tailored dietary modification goals and addiction-focused strategies (e.g., coping with withdrawal) would improve outcomes in behavioral interventions for obesity among individuals with UPF addiction, as well as identify individual characteristics that may inform whether an abstinence-based or harm reduction approach may be more optimal.

## Conclusion

The breadth of recent research on UPF addiction illustrates continued scientific and public interest in the construct and its implications for understanding and treating overeating behaviors and obesity. Topical developments have included: (1) estimating the global prevalence of UPF addiction at 14% of adults and 15% of youths (28% and 19% among samples with obesity, respectively); (2) revealing health disparities in rates of UPF addiction for persons of color and those with food insecurity; (3) observing altered functioning across the brain-gut-microbiome axis among individuals with UPF addiction; (4) providing early evidence for the plausibility of experiencing a withdrawal syndrome when cutting down on UPFs; and (5) elucidating poorer weight management outcomes among persons with UPF addiction. There are abundant next steps in this line of research, though a particularly pressing gap in the literature is the lack of targeted interventions for UPF addiction. Developing and demonstrating the efficacy of novel, addiction-based treatments for UPF addiction may result in more optimal clinical outcomes for this underserved population.

## Data Availability

No datasets were generated or analysed during the current study.
